# GREM1 is a potential biomarker for the progression and prognosis of bladder cancer

**DOI:** 10.1186/s12957-023-03128-0

**Published:** 2023-08-22

**Authors:** Peng-cheng Jiang, Li-zhe Xu, Jin-zhuo Ning, Fan Cheng

**Affiliations:** Department of Urology, Renmin Hospital of Wuhan University, Wuhan, Hubei Province People’s Republic of China

**Keywords:** GREM1, Bladder cancer, Progression, Apoptosis, PI3K/AKT signaling pathway

## Abstract

**Background:**

Gremlin-1 (GREM1) is a protein closely related to tumor growth, although its function in bladder cancer (BCa) is currently unknown. Our first objective was to study the GREM1 treatment potential in BCa.

**Methods:**

BCa tissue samples were collected for the detection of GREM1 expression using Western blot analysis and Immunofluorescence staining. Association of GREM1 expression with clinicopathology and prognosis as detected by TCGA (The Cancer Genome Atlas) database. The functional investigation was tested by qRT-PCR, western blot analysis, CCK-8, cell apoptosis, wound healing, and transwell assays. The interaction between GREM1 and the downstream PI3K/AKT signaling pathway was assessed by Western blot analysis.

**Results:**

GREM1 exhibited high expression in BCa tissues and was linked to poor prognosis. Stable knockdown of GREM1 significantly inhibited BCa cell (T24 and 5637) proliferation, apoptosis, migratory, invasive, as well as epithelial-mesenchymal transition (EMT) abilities. GREM1 promotes the progression in BCa via PI3K/AKT signaling pathway.

**Conclusion:**

Findings demonstrate that the progression-promoting effect of GREM1 in BCa, providing a novel biomarker for BCa-targeted therapy.

**Supplementary Information:**

The online version contains supplementary material available at 10.1186/s12957-023-03128-0.

## Introduction

Bladder cancer (BCa) is among the most widespread cancers worldwide, with expectations of 573,278 new cases and 212,548 mortalities in 2020. In addition, 25% of diagnosed patients had muscle-invasive BCa, which carries higher metastasis and mortality risks [1–3]. The primary treatment for muscle-invasive bladder cancer is a radical cystectomy associated with either neoadjuvant chemotherapy or followed by chemotherapy/immunotherapy. Also, trimodality treatment could be a choice in selected patients, which may enhance the prognosis for early-stage patients [4]. However, advanced BCa is still correlated to a poor prognosis, a significant likelihood of recurrence, and metastasis [5, 6]. Therefore, identifying the practical biomarkers is crucial for developing new and effective treatments for BCa [7, 8].

Gremlin-1 (GREM1) is an antagonist of bone morphogenetic protein (BMP) belonging to the cystine knot superfamily [9, 10]. GREM1 is crucial for bone formation, tissue differentiation, organ development, and embryogenesis [11, 12]. GREM1 is also linked to inflammation and organ fibrosis [13–16]. Yet, recent research has revealed that GREM1 is involved in the control of tumor growth. Previous research revealed a tight connection between GREM1 and the incidence and metastasis of numerous tumor types involving breast, lung, and prostate cancer [17]. Nevertheless, the functions of GREM1 in BCa development and the involved processes are yet unclear. We examined the roles and mechanisms behind GREM1 activation in BCa. Pictilisib is a potent and selective inhibitor of class I phosphatidylinositol-3-kinases (PI3K). The antitumor activity of Pictilisib was found to be more pronounced in bladder cancer patient-derived xenograft (PDX) models with mutations or amplifications in the PI3K gene compared to the control PDX model [18]. Zhu et al. [19] found that PI3K inhibition inhibited cell proliferation, migration, and colony formation in bladder cancer cells. Pan-PI3K inhibition increased overall survival (OS) from 27 to 48 days in syngeneic mice with PTEN-deleted tumors. Knockdown of GREM1 inhibited BCa progression via PI3K/AKT signaling pathway. Therefore, GREM1 could be utilized as a biomarker for detecting and treating BCa.

## Materials and methods

### Tissue specimens

BCa tissues, as well as nearby healthy tissues, were retrieved from BCa patients who underwent radical cystectomy at Renmin Hospital of Wuhan University. We used liquid nitrogen to freeze tissue samples. Each patient gave their written approval to have their healthcare records utilized for research by signing the consent form. Renmin Hospital of Wuhan University Ethics Committee approved this research (approval number. WDRY2019-K035).

### Bioinformatic analysis

In addition to medical history, TCGA (The Cancer Genome Atlas) database (https://portal.gc.cancer.gov/) was utilized to extract RNA-seq gene expression data. Data formatting for level 3 HTSeq fragments per kilobase per million was obtained. GREM1 contents in 33 kinds of human malignancies, 412 BCa with 19 non-malignant tissues, and 19 BCa alongside matched nearby non-malignant tissues were investigated Utilizing the TCGA database. For differentially expressed genes (DEGs) identification, a |log2 fold change (FC)|> 1 and adjusted *p* values less than 0.05 were regarded as the threshold. Using the ggplot2 package in R, a correlation study of GREM1 and cancer stage, comprising T, N, pathological, and histologic stages, was conducted. The Kaplan–Meier plots and the log-rank test were constructed with R utilizing the survival package. Correlation measures were evaluated via the R package function cor.test utilizing the Pearson method. Genes with the highest-ranking positive or negative correlation coefficients with GREM1 were chosen. The clusterProfiler software did Gene Ontology (GO) analysis with the Kyoto Encyclopedia of Genes and Genomes (KEGG) enrichment analysis.

### Immunofluorescence staining

First, tissue samples were fixed in formalin, embedded in paraffin, and sliced into 4 μm thickness. After deparaffinization, tissue pieces were rehydrated. After 0.3% hydrogen peroxide in methanol treatment, slices were blocked in 1% BSA for 30 min. Then slices incubation was done at 4 °C overnight with Anti-GREM1 (cat. no. PK12868; 1:100; Abmart) at 4 °C overnight. Slices were carefully cleaned using PBS, then treated for 30 min with DAB and horseradish peroxidase (HRP)-conjugated IgG. Finally, the slices were hematoxylin stained again and imaged under a microscope (Olympus BX53).

### RNA extraction and quantitative reverse transcription-polymerase chain reaction (qRT-PCR)

Utilizing the TRIzol reagent, total RNA extraction was done (Invitrogen; Thermo Fisher Scientific). Reverse transcription was conducted for cDNA generation with a Takara reagent kit (Takara Biotechnology, Otsu, Japan). qPCR amplification was then done utilizing SYBR Green Master Mix (Yeasen, Shanghai, China) as directed by the manufacturer. In this study, the following primers were utilized: GREM1, forward primer sequence: 5′-GTCACACTCAACTGCCCTGA-3′ and reverse primer sequence: 5′-GGTGAGGTGGGTTTCTGGTA-3′; GAPDH, forward primer sequence: 5′-GTGGACCTGACCTGCGTCT-3′ and reverse primer sequence: 5′-GTGTCGCTGTTGAAGTCAGAGGAG-3′.

### Western blot analysis

Total protein samples were retrieved from bladder tissues or cultured cells, and their quantity was assessed by a BCA Protein Assay Kit (Invitrogen) after processing in RIPA lysis buffer supplied with protease and phosphatase inhibitors [20, 21]. For western blot analysis, a 10% SDS-PAGE gel and 30 μg of proteins were packed and transported to a PVDF membrane. Next, the membranes were blocked for 1 h at room temperature with 5% milk before incubation with primary antibodies: anti-GREM1 (#PK12868; 1:1000; Abmart), anti-E-Cadherin (#A20798; 1:1000; Abclonal), anti-N-cadherin (#A19083; 1:1000; Abclonal), anti-Vimentin (#A19607; 1:1000; Abclonal), anti-p-PI3K (#17366; 1:1000; CST), anti-PI3K (#T40064; 1:1000; Abmart), anti-p-AKT (#4060; 1:1000; CST), anti-AKT (#60203; 1:5000; Proteintech) or anti-GAPDH (#AC001; 1:5000; Abclonal) at 4 °C overnight. The following day, the membranes were incubated with peroxidase-conjugated secondary antibody at room temperature for 1 h, pictured by the chemiluminescence system (ChemiDocTM Touch; Bio-Rad, USA), and assessed with the ImageJ program.

### Cell lines and culture

T24 and 5637 human BCa cell lines were acquired from the Chinese Academy of Sciences Cell Bank (Shanghai, China). T24 and 5637 cells were grown in RPMI-1640 culture media (HyClone; Cytiva, USA). All cell cultures were supplied with 10% fetal bovine serum (Hangzhou Sijiqing Biological Engineering Materials Company, Hangzhou, China) and 1% penicillin/streptomycin (Gibco; Thermo Fisher Scientific, United States) and cultured in a humidified incubator with 5% CO_2_ at 37 °C.

### Cell transfection

Genechem Corporation (Shanghai Province, China) provided knockdown lentivirus for GREM1 (named shGREM1) and control lentivirus (named shNC). Cells seeding was done in 6-well plates at a density of 1 × 10^6^ cells/well and transfected after achieving 50% confluence based on instructions of the manufacturer. The 5637 and T24 cell lines transfection, was done with shGREM1 or shNC. Cells were treated with 2 μg/mL of puromycin to obtain stably transfected cell lines. Stably transfected cells were utilized in the following investigations.

### CCK-8 cell proliferation assay

Cell viability was tested by CCK-8 Assay Kit (Biosharp, Hefei, China). Cells were inoculated in 96-well plates at a density of 1 × 10^3^ cells/well, transfected for 0, 24, 48, and 72 h, and stained with the CCK-8 reagent for 2 h at 37 °C. With a microplate reader, absorbance was measured at 450 nm to estimate cell viability (Ensight; Perkin Elmer, Waltham, MA, USA).

### Cell apoptosis assay

Treated T24 and 5637 cells were trypsin digested without EDTA, then washed with cold PBS, and resuspended in buffer at a density of 5 × 10^5^ cells/mL. Cells were stained twice with Annexin V/PI Apoptosis Detection Kit (Elabscience) as directed by the manufacturer and let to sit for 30 min at room temperature. Later, stained cell detection was conducted utilizing flow cytometry (CytoFlex; Beckman Coulter Life Sciences).

### Wound healing assay

Each group's transfected T24 and 5637 cells were seeded and cultured to 80–90% confluence in six-well plates. Thereafter, a sterile 200 μL pipette tip was employed for scratching the cell monolayer. The plates were then grown in serum-free media after being cleaned twice or three times with PBS in each well. A light microscope (Olympus IX71; Olympus Corp., Tokyo, Japan) was utilized to capture the width of wound at 0 and 24 h, and the cell migration area (μm^2^) was measured utilizing ImageJ software.

### Cell migration and invasion assays

Using 6-well Transwell plates (Corning) and 8-m-pore membranes containing matrix gel (for invasion testing) or without matrix gel, cell migration and invasion were investigated (for migration testing). T24 and 5637 cells were obtained 48 h following transfection. Briefly, cells were planted in the matrix gel-covered upper chamber, and a 10% fetal calf serum-containing media was introduced to the lower chamber. Following incubation, the top lumen membrane cells were washed away. Stained using 0.2% crystal violet for 30 min, then counting five predefined fields under a microscope. Inserts were covered with Matrigel matrix (BD Science, Sparks, MD, USA) diluted in serum-free media and incubated at 37 °C for 2 h for invasion analysis, and the rest of procedures were performed similarly to migration analysis.

### Statistical analysis

GraphPad Prism 8 (GraphPad Software, La Jolla, CA, USA) was used for the statistical analysis of data. Findings are provided as mean ± SD for every group. For statistical analysis, the Student's t-test and one-way analysis of variance (ANOVA) were employed. *p* less than 0.05 was set as a statistical significance.

## Results

### GREM1 displays a higher expression level in cancer tissues versus control tissues

**Fig. 1:**
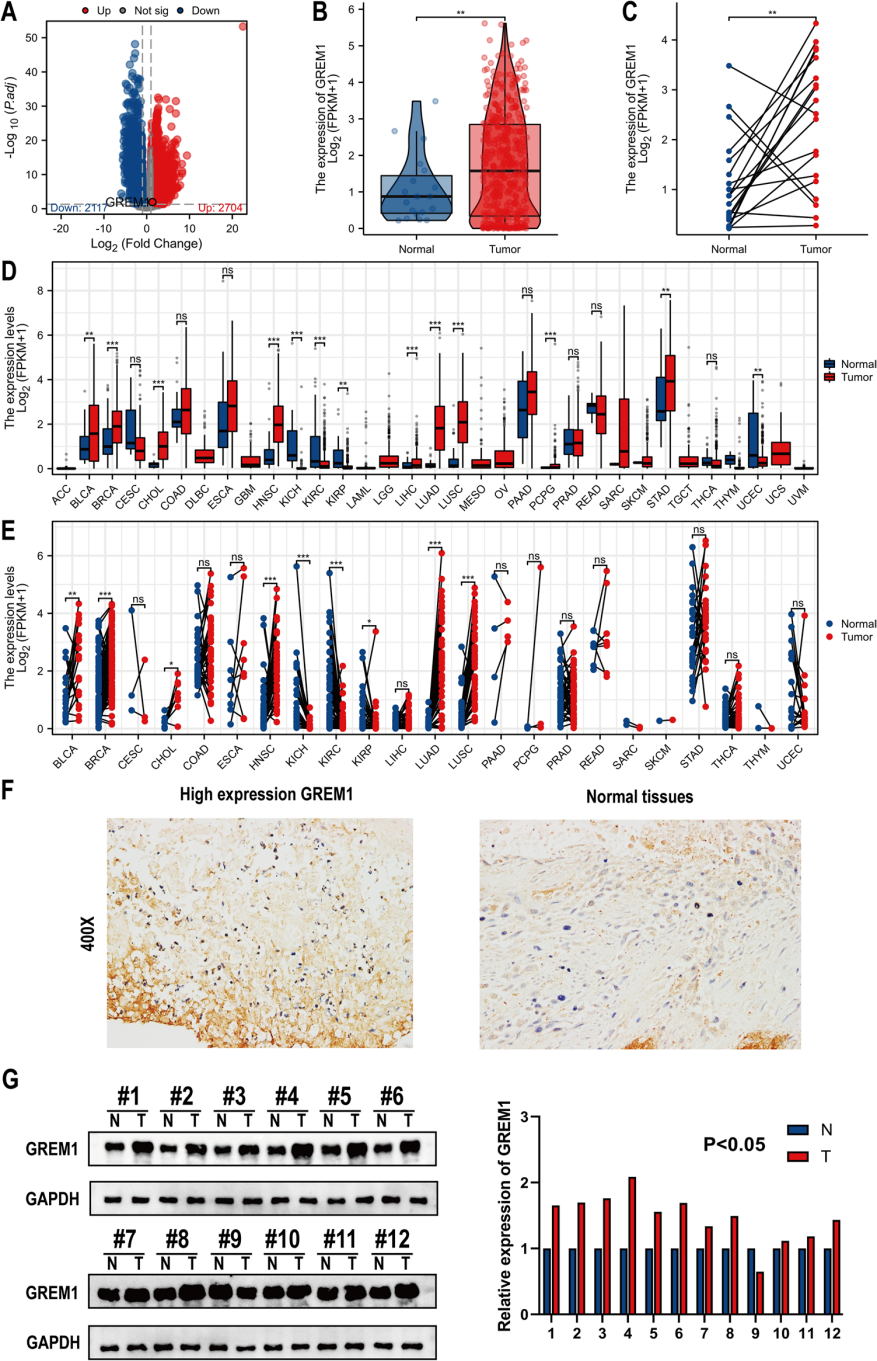
GREM1 expression characteristics analysis. **A** Volcano plot of the DEGs. The red and blue dots represent the significantly upregulated and significantly down-regulated genes in BCa. **B** The difference expression of GREM1 in BCa tissues and adjacent normal tissues. **C** The difference expression of GREM1 in BCa tissues and paired normal tissues. **D** GREM1 expression profile across multiple cancer samples and normal tissues. **E** GREM1 expression profile across multiple cancer samples and paired normal tissues. **F** Representative immunohistochemistry of GREM1 in BCa tissues and adjacent normal tissues. **G** Western blot of GREM1 protein expression in BCa tissues and adjacent normal tissues. *p < 0.05, **p < 0.01, ***p < 0.001

Among 18,814 genes, a sum of 4821 DEGs was screened (2704 upregulated and 2117 downregulated) utilizing the adjusted *p* value < 0.05 along with |log2FC|> 1 threshold. The DEGs expression distributions are shown as a volcano graphic (Fig. 1A). Further, we discovered that GREM1 was significantly increased in tumor tissues than in nearby healthy bladder tissues (Fig. 1B, C). The findings indicated that GREM1 expression was significantly increased in the majority of malignancies in contrast to corresponding control tissues (Fig. 1D, E). A sum of 28 pairs of BCa and nearby healthy tissues were gathered for further investigations. Findings of IHC performed on these samples revealed an elevated expression of GREM1 in BCa tissues (Fig. 1F). Western blot analysis revealed a dramatic elevation in GREM1 protein level in BCa samples compared to paired control bladder tissues (Fig. 1G). Our results suggested that GREM1 exhibits higher levels of expression in BCa in comparison with normal tissues, indicating that it might have a significant role in tumor progression.

### GREM1 expression is associated with BCa progression and prognosis

**Fig. 2:**
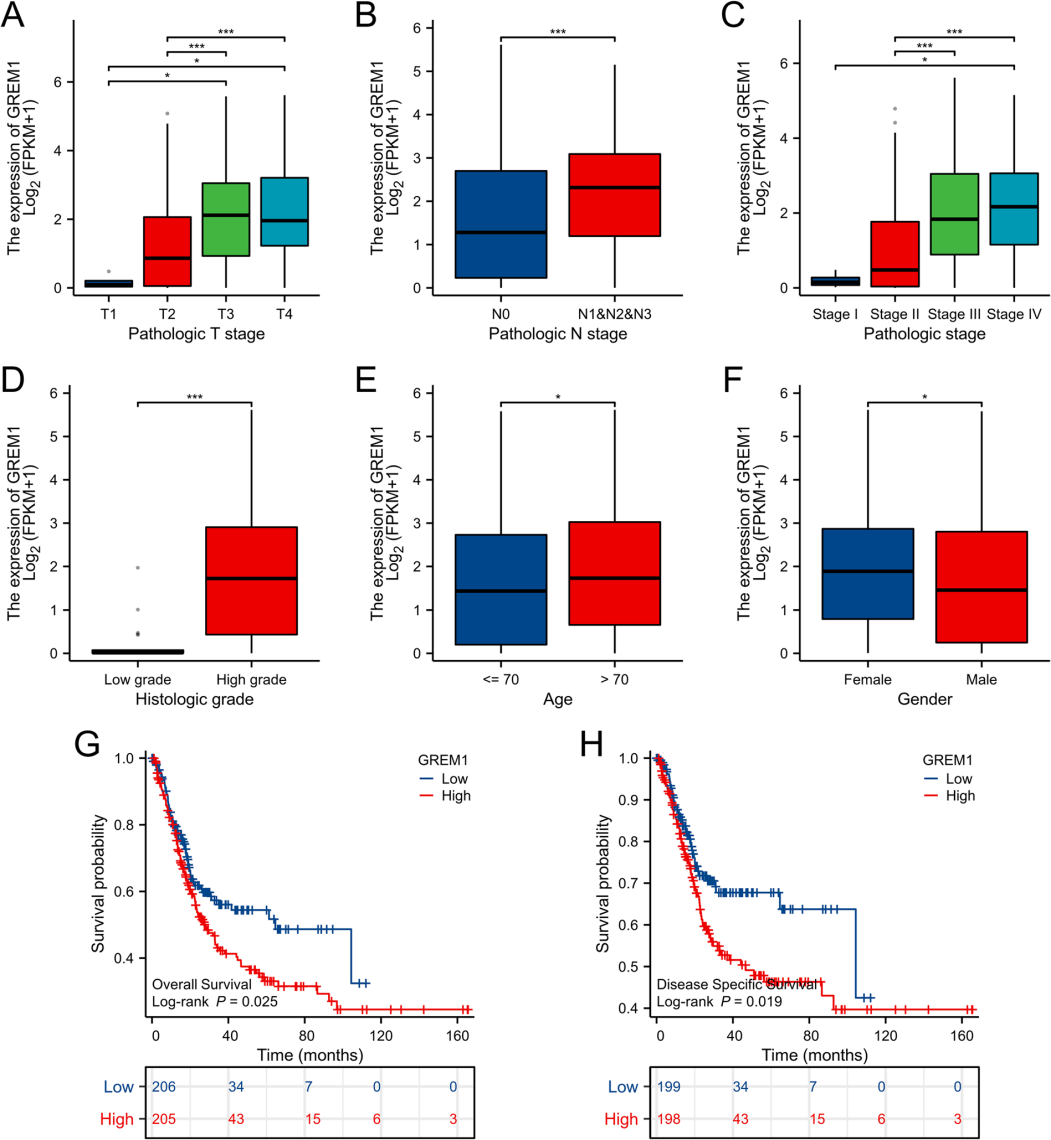
The correlation with clinicopathological features of GREM1 gene in BCa. **A-F** Relative expression levels of GREM1 in TCGA database with T stage, N stage, pathological stage, histologic grade, age, gender. **G, H** OS and DSS curve of BCa patients with low and high GREM1 expression in TCGA database. *p < 0.05, ***p < 0.001

The findings of our research on the TCGA database revealed that GREM1 expression is linked with the T stage, N stage, and pathological stage when we looked at the association between GREM1 and clinical characteristics. The expression of GREM1 exhibited a gradual increase as the T stage, N stage, and pathological stage progressed for BCa patients (Fig. 2A–C). In addition, we showed that histologic grade (Fig. 2D), age (Fig. 2E), and gender (Fig. 2F) with GREM1 expression were independent factors for BCa patients. Based on the Kaplan–Meier survival curves, BCa patients with higher GREM1 levels exhibited worse overall survival (OS) (Fig. 2G) and lower disease-specific survival (DSS) (Fig. 2H).

### GREM1 knockdown inhibits proliferation and promotes apoptosis of BCa cells in vitro

**Fig. 3:**
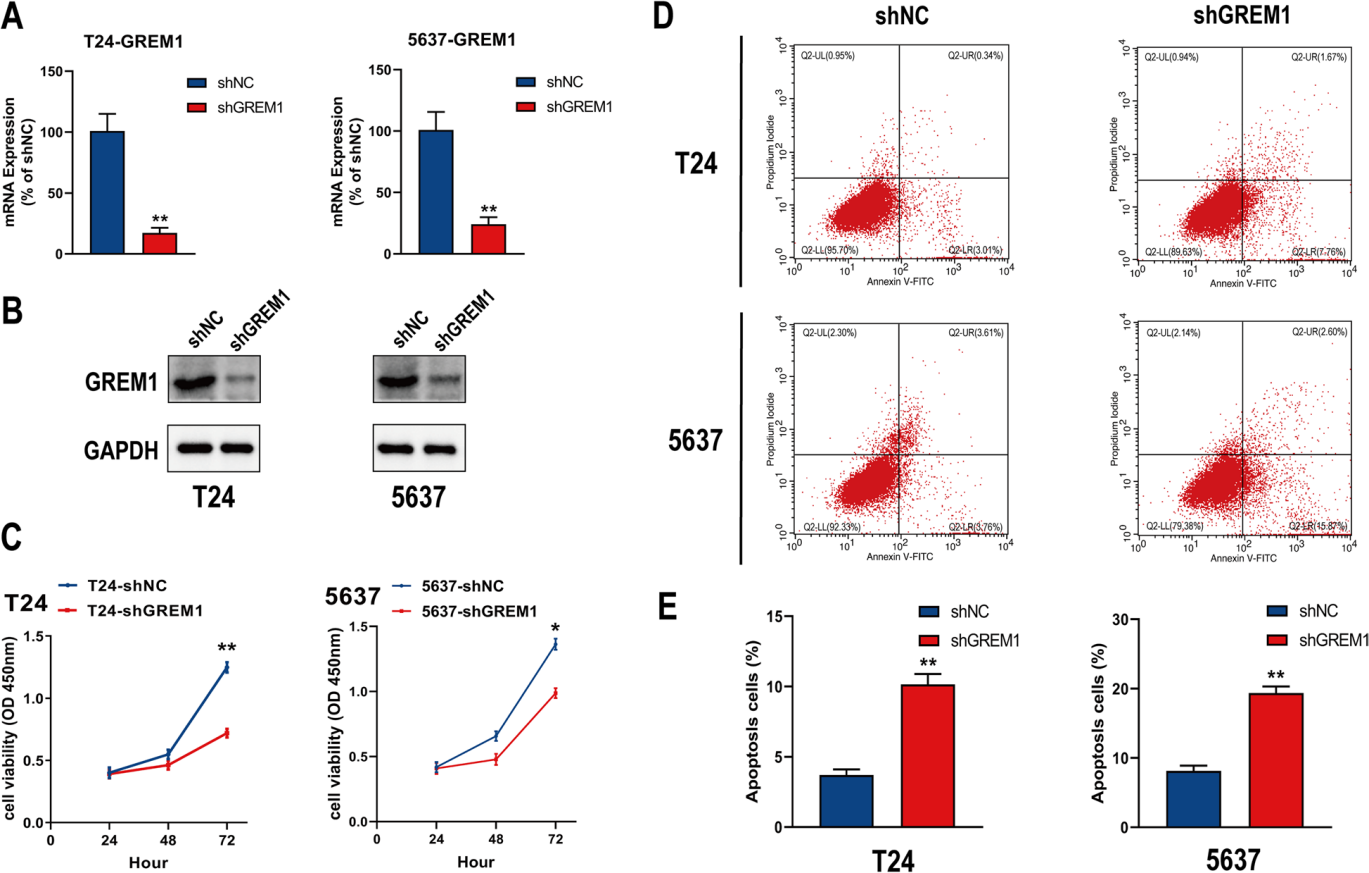
Knockdown of GREM1 inhibits the proliferation and enhances apoptosis of BCa cells in vitro. **A**,** B** The knockdown efficiency of GREM1 was confirmed by qRT–PCR and WB analysis. **C** Effects of GREM1 knockdown on cell proliferation by CCK-8 assays in T24 and 5637 cells. **D**,** E** Effects of GREM1 knockdown on cell apoptosis by flow cytometry in T24 and 5637 cells. *p < 0.05, **p < 0.01

We generated the GREM1 defective cell models for assessment of the impact of GREM1 on the growth and apoptosis of BCa cells. GREM1 mRNA expression was significantly suppressed by 93% in T24 and 85% in 5637 after transfection of BCa cells with knockdown lentivirus and control lentivirus, respectively (Fig. 3A). The knockdown lentivirus consistently drastically reduced the protein level of GREM1 (Fig. 3B). GREM1 silencing could reduce the viability of T24 and 5637 cells, according to the CCK-8 experiment (Fig. 3C). In addition, when GREM1 was knocked down in BCa cells in comparison to the shNC group, flow cytometry showed a markedly increased proportion of apoptotic cells (Fig. 3D, E). Our findings demonstrated that suppression of GREM1 inhibited BCa cell growth while increasing BCa cells apoptosis.

### GREM1 knockdown inhibits BCa cell migration, invasion, and EMT in vitro

**Fig. 4:**
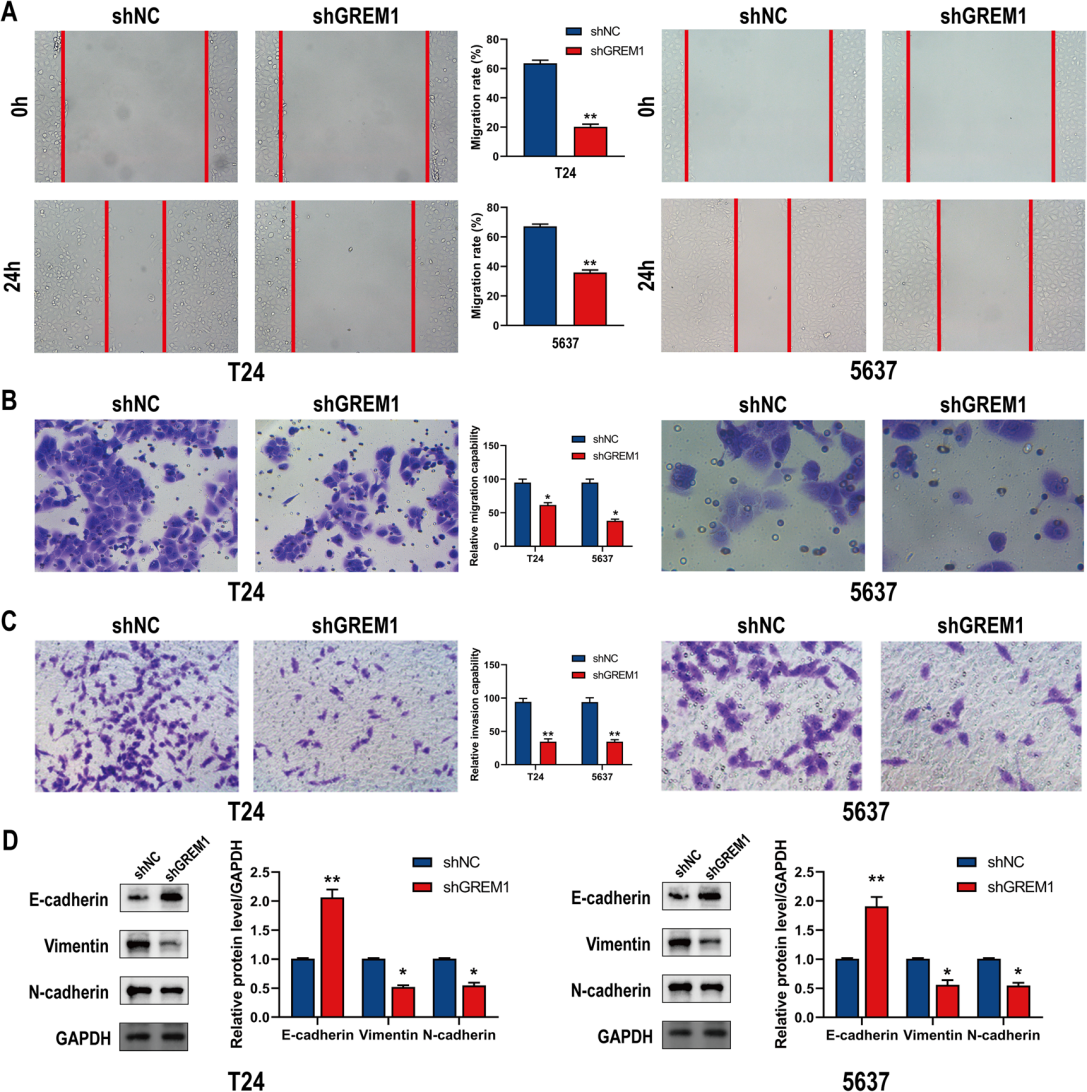
Knockdown of GREM1 inhibits the migration, invasion and EMT of BCa cells in vitro. **A** Effects of GREM1 knockdown on migration by wounding healing assays in T24 and 5637 cells. **B** Effects of GREM1 knockdown on migration by transwell assays in T24 and 5637 cells. **C** Effects of GREM1 knockdown on invasion by transwell assays in T24 and 5637 cells. **D** Changes in the EMT-related proteins were detected by Western blot in T24 and 5637 cells. * *p* < 0.05, ** *p* < 0.01

Epithelial-mesenchymal transition (EMT) has been linked to carcinogenesis and gives metastatic qualities to cancer cells by improving their motility, invasion, and apoptosis resistance [22]. The impact of GREM1 knockdown on BCa cell motility was measured using a wound-healing assay. GREM1 knockdown significantly reduced T24 and 5637 cell motility (Fig. 4A). Similarly, transwell assays with or without matrix gel showed that T24 and 5637 cell invasiveness was observably reduced by GREM1 knockdown (Fig. 4B, C). Western blotting analysis suggested that for both BCa cell lines, GREM1 knockdown may result in a considerable overexpression of E-ca and a significant downregulation of N-ca and Vimentin (Fig. 4D).

### GO together with KEGG pathway enrichment analysis of GREM1 co-expression genes

**Fig. 5:**
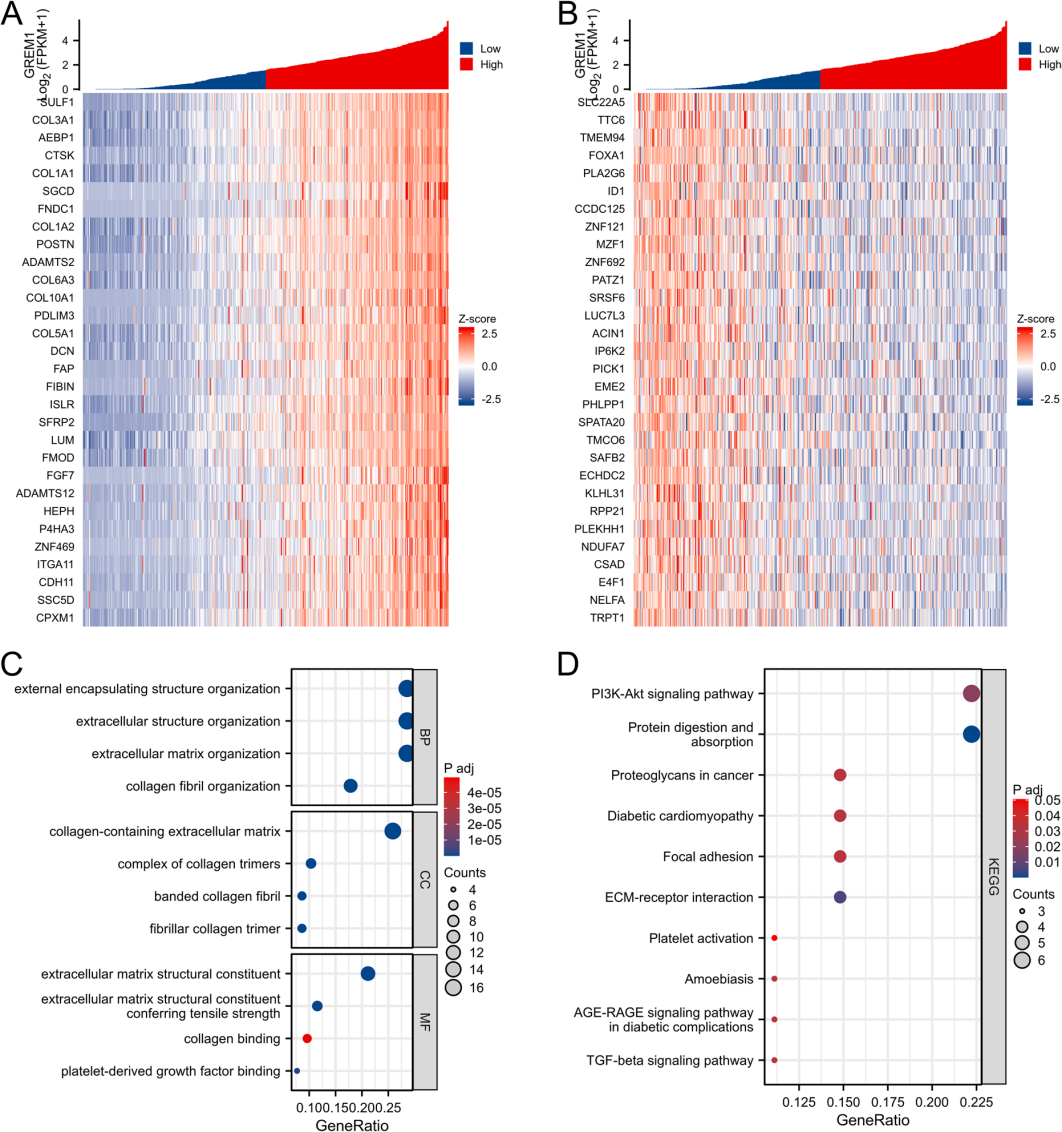
GREM1 co-expressed genes and functional enrichment analysis in TCGA-BLCA patients. **A**,** B** Top 30 positively and top 30 negatively correlated genes with GREM1 in BCa are illustrated in the heatmap. **C-D** GO and KEGG pathway analyses of top 30 positively and top 30 negatively correlated genes with GREM1 in BCa

To understand the biological functions of GREM1 in BCa, we detect the co-expression genes of GREM1 in BCa using data from the TCGA database. The top 30 genes exhibited a positive, and the top 30 genes exhibited a negative correlation with GREM1 in BCa, which are illustrated in the heatmap (Fig. 5A, B). To comprehensively understand the potential functions and molecular mechanisms, we carried out GO and KEGG pathway analysis for top 30 positively and top 30 negatively correlated genes with GREM1 in BCa. The PI3K/AKT signaling pathway and GREM1 expression are substantially positively correlated based on KEGG pathway analysis (Fig. 5C, D).

### GREM1 modulated the PI3K/AKT signaling pathway

**Fig. 6:**
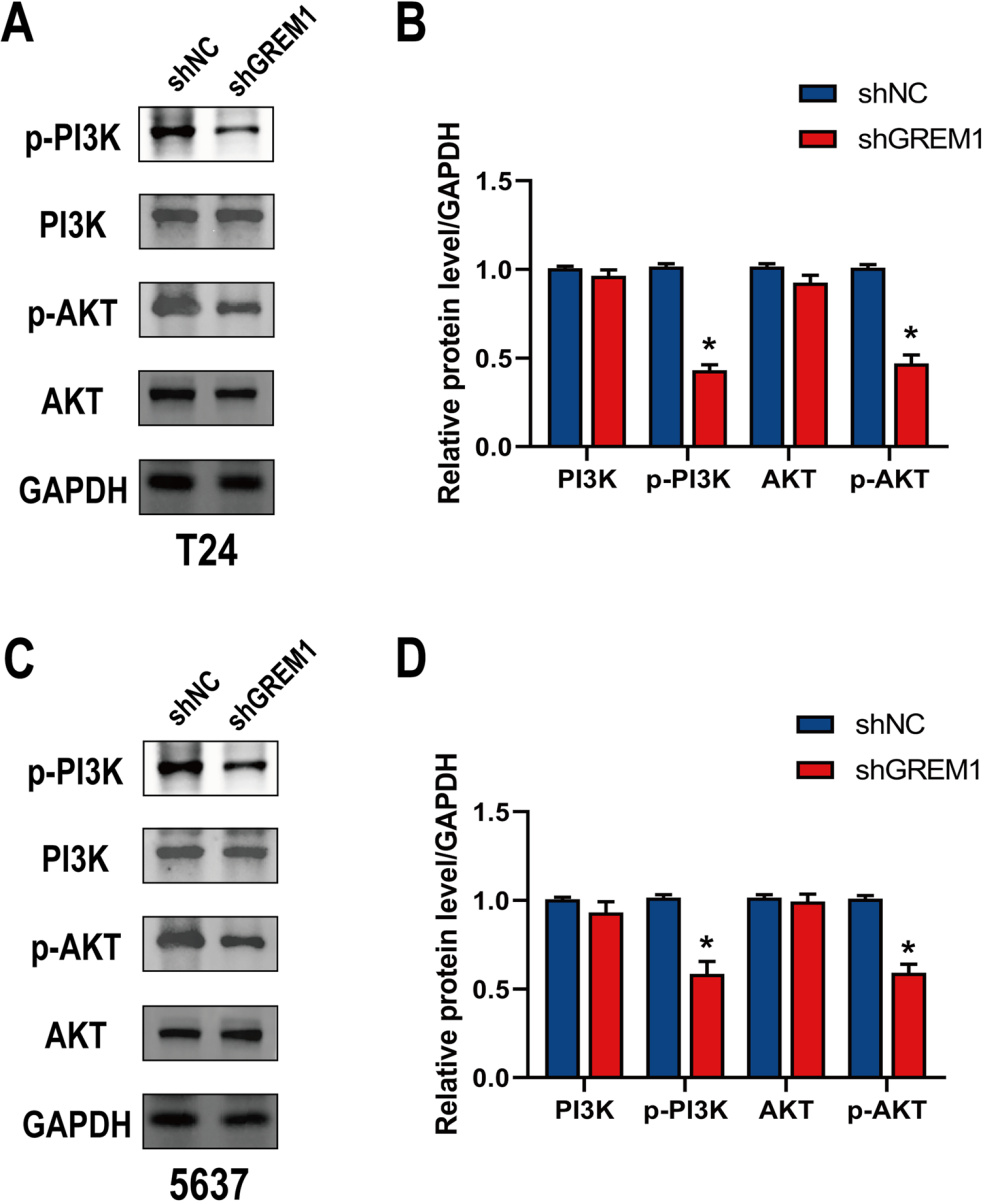
Knockdown of GREM1 inhibits migration, invasion and EMT by regulating the PI3K/AKT signaling pathway in BCa cells. **A**,** B** Representative western blot images and quantitative analyses of related proteins, including p-PI3K/PI3K and p-AKT/AKT in T24 cells. **C**,** D** Representative western blot images and quantitative analyses of related proteins, including p-PI3K/PI3K and p-AKT/AKT in 5637 cells. * *p* < 0.05

Cancers often exhibit abnormalities in the primary components of PI3K/AKT signaling pathway [23]. The phosphorylation of PI3K(p-PI3K) and AKT(p-AKT) protein levels was significantly reduced in GREM1 deficient T24 and 5637 cells, as determined by Western blotting (Fig. 6A–D). Our findings suggest that GREM1 could take part in BCa development through PI3K/AKT signaling pathway, as forecasted by KEGG analysis.

## Discussion

Localized or metastatic BCa therapy has undergone substantial transformations in the last two decades due to notable progress in combination chemotherapy and immunotherapy. A rising number of studies on bladder cancer suggests that the progression of this malignancy is primarily caused by alterations in molecular mechanisms [24]. For a more thorough diagnosis and therapy of bladder cancer, it was essential to identify the proper molecular targets. Park et al. [11] found a relationship between GREM1 levels and patients with breast cancer survival rates. Cheng et al. [25] found that GREM1 promotes lineage plasticity and drives castration resistance in prostate cancer. Kan et al. [26] showed that GREM1 upregulation was closely related to tumor metastasis in lung cancer. OE-GREM1 promoted tumor cell migration, invasion, and EMT. Davis et al. [27] demonstrated that GREM1 disrupts homeostatic intestinal morphogen gradients and can initiate human colorectal cancer. Yet, no research has been done to determine whether GREM1 contributed to the development of BCa. In this work, we determined that GREM1 is an important latent molecular target for diagnosing and treating BCa.

GREM1, a BMP antagonist that may play an important role during carcinogenesis and organogenesis, was discovered as being highly expressed in BCa tissues in both the TCGA dataset as well as clinical samples. Except for gene abnormality, other risk factors for BCa include age, gender, smoking, occupational exposure, infections, and dietary factors (meat, tea, and vegetables) [28–30]. In this research, first, the expression and prognostic significance of GREM1 protein in the TCGA BCa dataset were assessed. Our investigation revealed substantial expression of GREM1 in BCa tissues. GREM1 expression was favorably connected with the TN stage, grade, and pathological stage but was adversely correlated with OS and DSS. We discovered that the knockdown of GREM1 decreased BCa cell proliferation, migration, invasion, and EMT processes while promoting apoptosis in vitro functional experiments. Mechanistically, we showed that GREM1 might contribute to the growth and proliferation of BCa through the PI3K/AKT signaling pathway.

Treatment and prognosis are impacted by invasion and distant metastases in BCa patients [31]. For patients with metastatic uroepithelial carcinoma, platinum-based chemotherapy is recommended as the first-line standard of care, provided they are suitable for treatment with cisplatin or carboplatin. Patients positive for programmed death ligand 1 (PD-L1) and ineligible for cisplatin may receive immunotherapy (atezolizumab or pembrolizumab). In case of nonprogressive disease on platinum-based chemotherapy, subsequent maintenance immunotherapy (avelumab) is recommended. For patients without maintenance therapy, the recommended second-line regimen is immunotherapy (pembrolizumab). Later-line treatment has undergone recent advances: the antibody–drug conjugate enfortumab vedotin demonstrated improved overall survival and the fibroblast growth factor receptor (FGFR) inhibitor erdafitinib appears active in case of FGFR3 alterations [32]. With the EMT process, BCa cells often increase their capacity to infiltrate into nearby tissues, which promotes tumor growth [33]. Research revealed that the migration and invasion capability of BCa cells was considerably reduced when GREM1 expression was knocked down. When GREM1 was silenced, the epithelial marker E-ca was overexpressed, whereas the interstitial markers N-ca and Vimentin were downregulated.

The PI3K/AKT signaling pathway is triggered or changed by cancer type and controls a wide variety of cellular activities, such as survival, proliferation, metabolism, angiogenesis, and metastasis [23, 34, 35]. Recently, drugs and molecules have been used to target this signaling pathway to treat cancer, with encouraging results in vitro, in vivo, and clinical trials. Duvelisib is a novel and selective PI3K δ/γ inhibitor, a global phase 3 randomized study of duvelisib vs ofatumumab monotherapy for patients with relapsed or refractory (RR) chronic lymphocytic leukemia (CLL)/small lymphocytic lymphoma (SLL) showed that duvelisib significantly improved progression-free survival per compared with ofatumumab for all patients (median, 13.3 months vs 9.9 months; hazard ratio [HR] = 0.52; *P* < 0.0001), including those with high-risk chromosome 17p13.1 deletions and/or TP53 mutations (HR = 0.40; *P* = 0.0002) [36]. Capivasertib is a potent inhibitor of pan-AKT kinase. In a phase 3 randomized, double-blind trial of patients with hormone receptor-positive, human epidermal growth factor receptor 2-negative advanced breast cancer who recurred or progressed on treatment, median progression-free survival was extended from 3.1 months to 7.2 months for patients receiving capivasertib plus fulvestrant compared to patients receiving placebo plus fulvestrant [37]. The significance of this pathway as a therapeutic target in BCa is mostly attributable to its major role in disease origin and progression [38]. Our research revealed that the expression of phosphorylated PI3K and phosphorylated AKT was considerably decreased when GREM1 was knocked down.

## Conclusion

All in all, we established for the first time that GREM1 is elevated in BCa tissues and that a high level of GREM1 expression is strongly correlated to a high pathological grade of BCa in patients. In addition, GREM1 is a major factor in cell proliferation, migration, and invasion in BCa. Moreover, PI3K/AKT signaling pathway activation is linked to GREM1 oncogenic function in BCa. The GREM1/PI3K/AKT axis could offer not only a new clinical biomarker for predicting the prognosis but also an excellent prospective therapeutic target for metastatic BCa.

### Supplementary Information

**Additional file 1.**

## Data Availability

The data that support the findings of this study are available from the corresponding author upon reasonable request.
