# Supplementary material for: GREM1 is a potential biomarker for the progression and prognosis of bladder cancer
Source: World J Surg Oncol. 2023 Aug 22;21:255. doi: 10.1186/s12957-023-03128-0 (PMC10463405; doi:10.1186/s12957-023-03128-0)

Figure 1

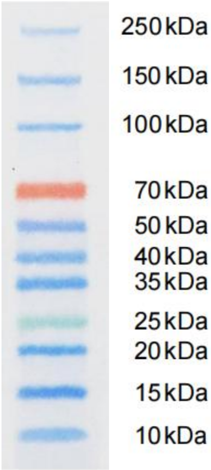

anti-GREM1-**23kDa**

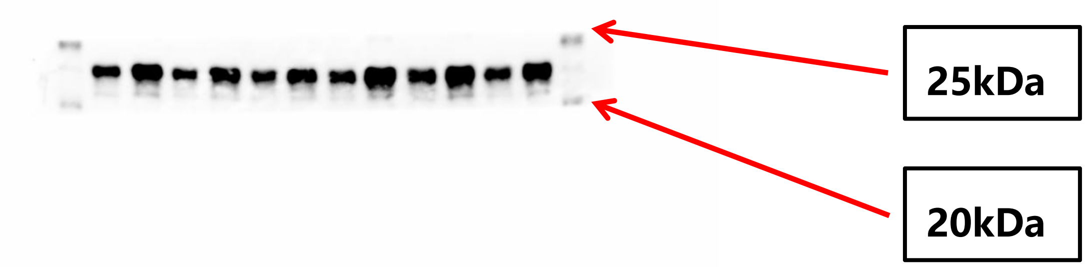

anti-GAPDH-**36KDa**

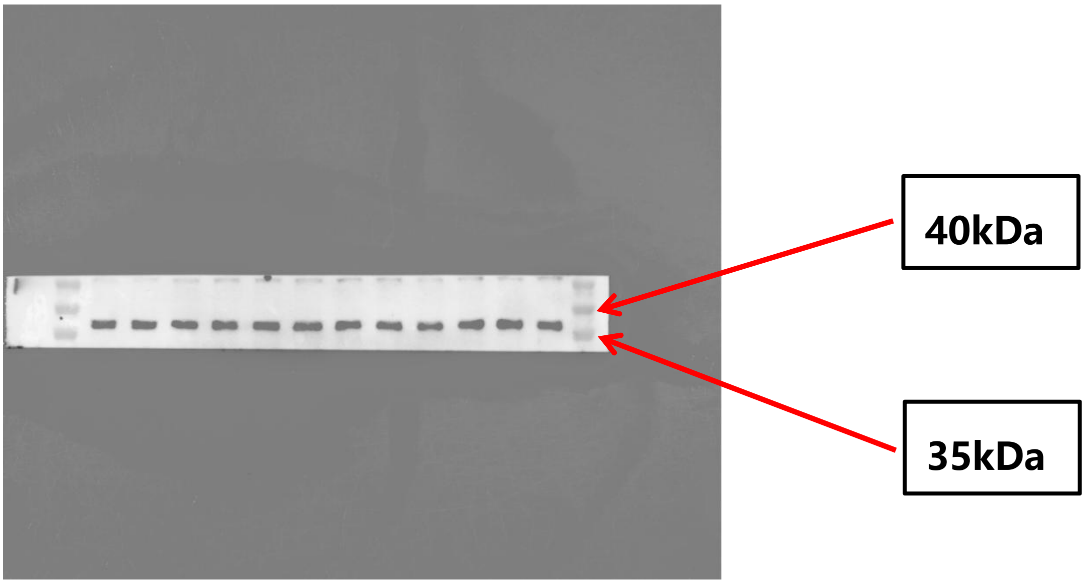

anti-GREM1-**23kDa**

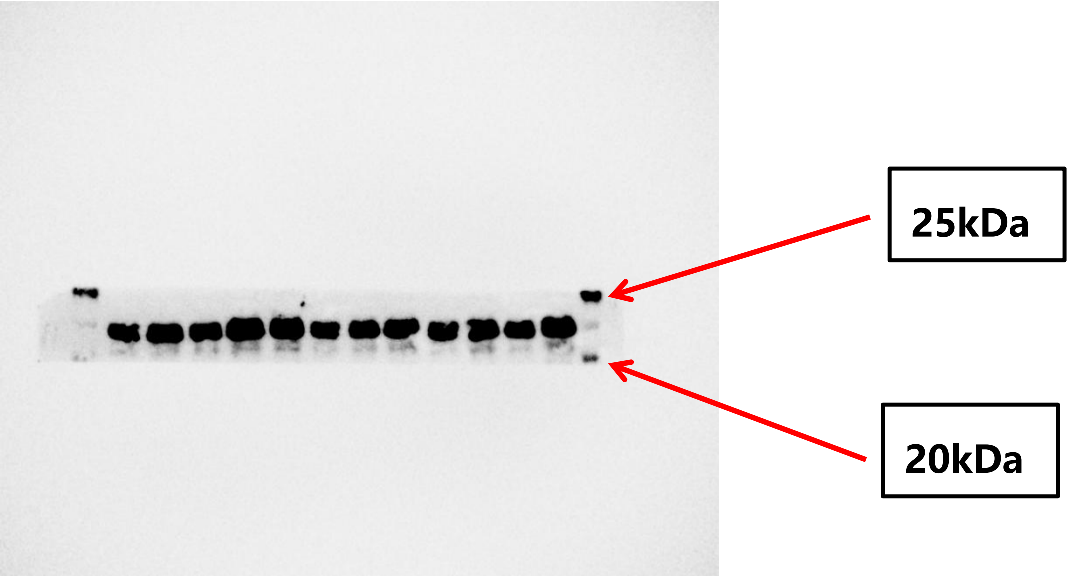

anti-GAPDH-**36KDa**

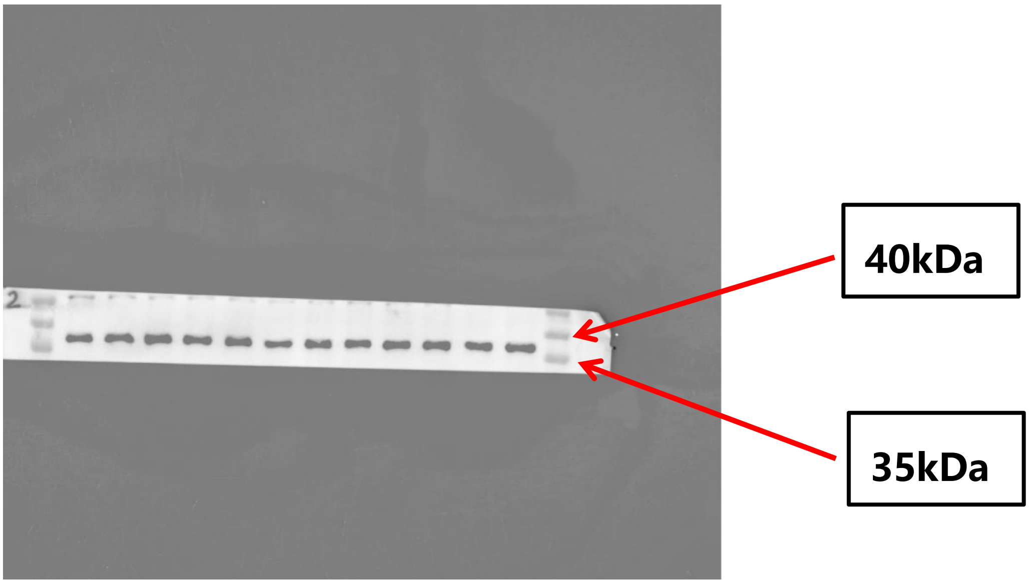

Figure 3

anti-GREM1**-23kDa**

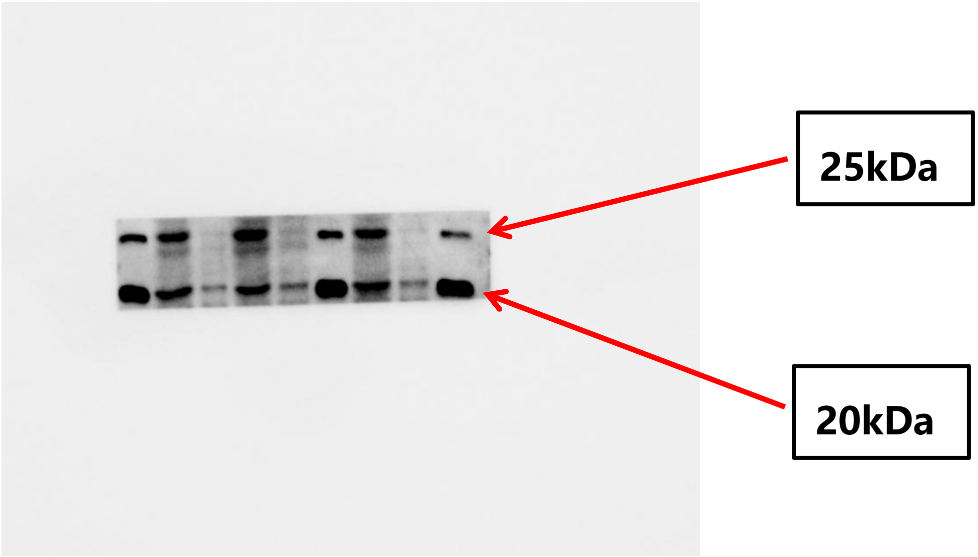

anti-GAPDH**-36KDa**

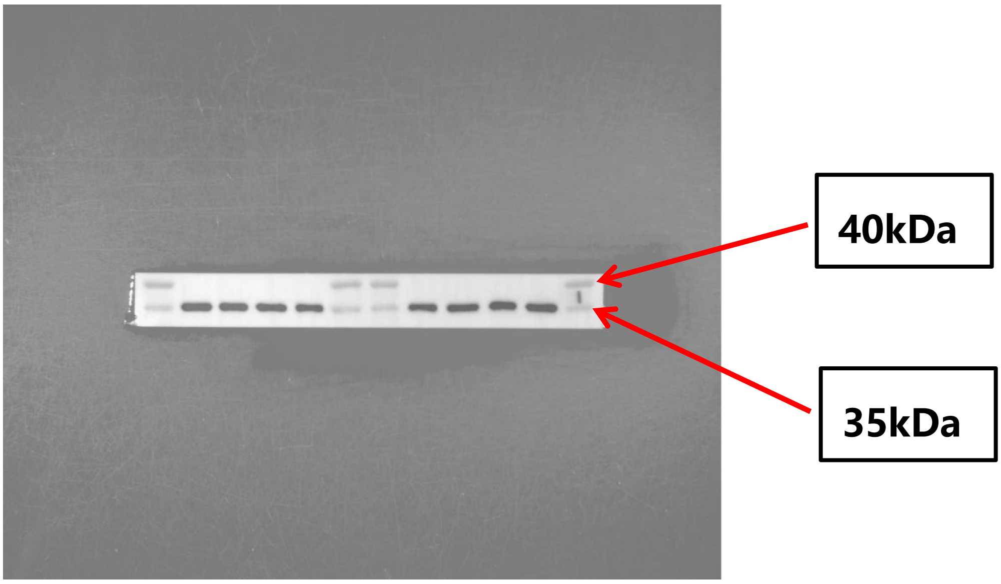

Figure 4

anti-E-Cadherin-**135KDa**

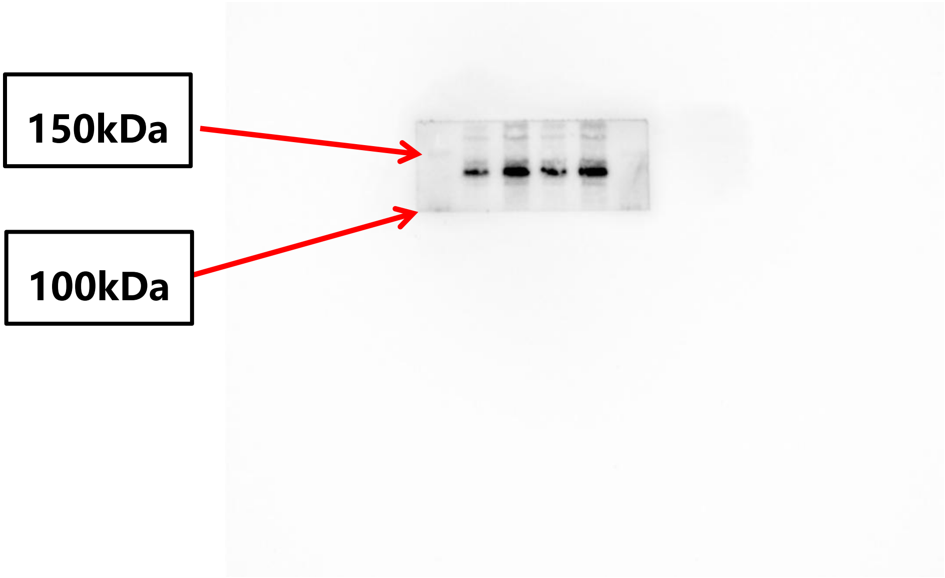

anti-Vimentin-**54kDa**

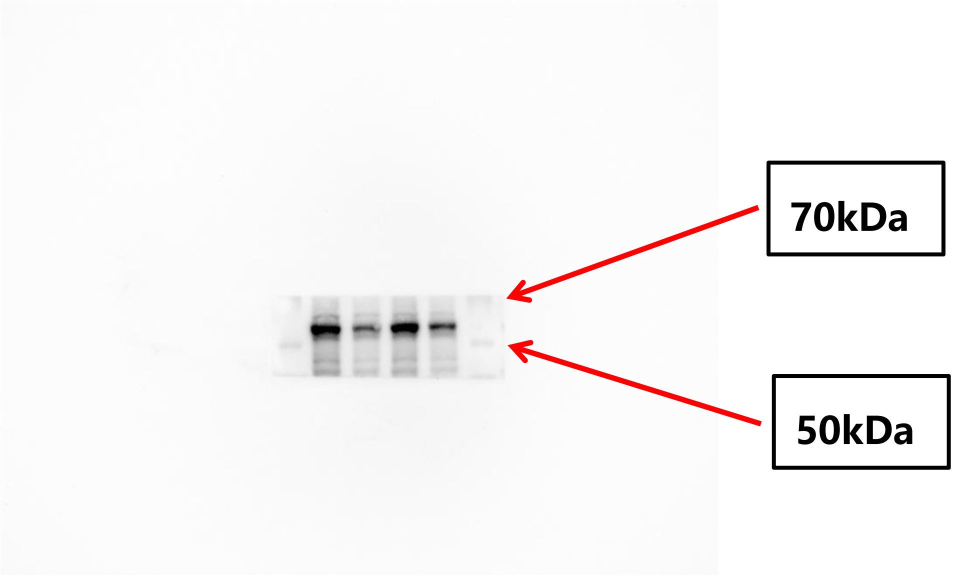

anti-N-Cadherin-**135KDa**

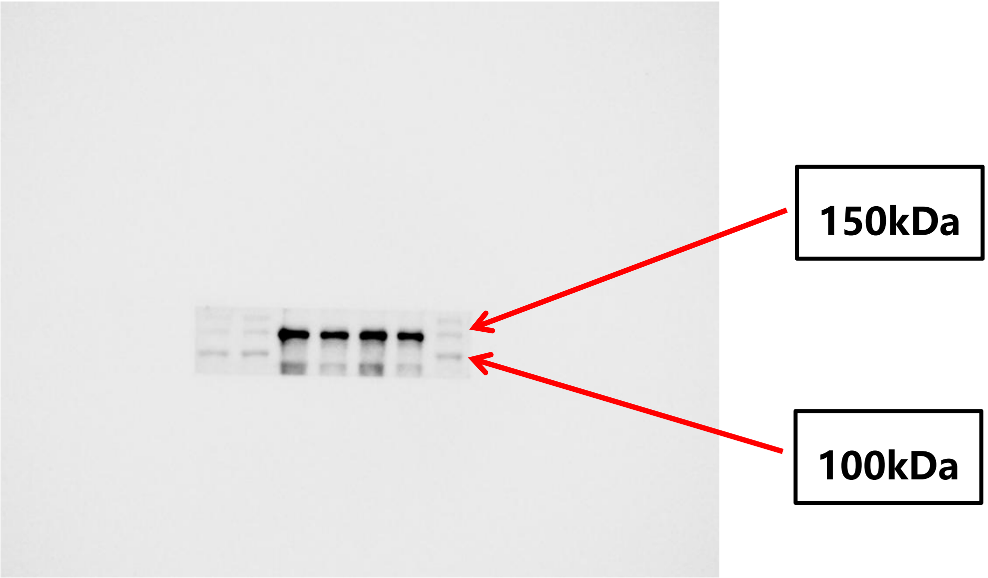

anti-GAPDH-**36KDa**

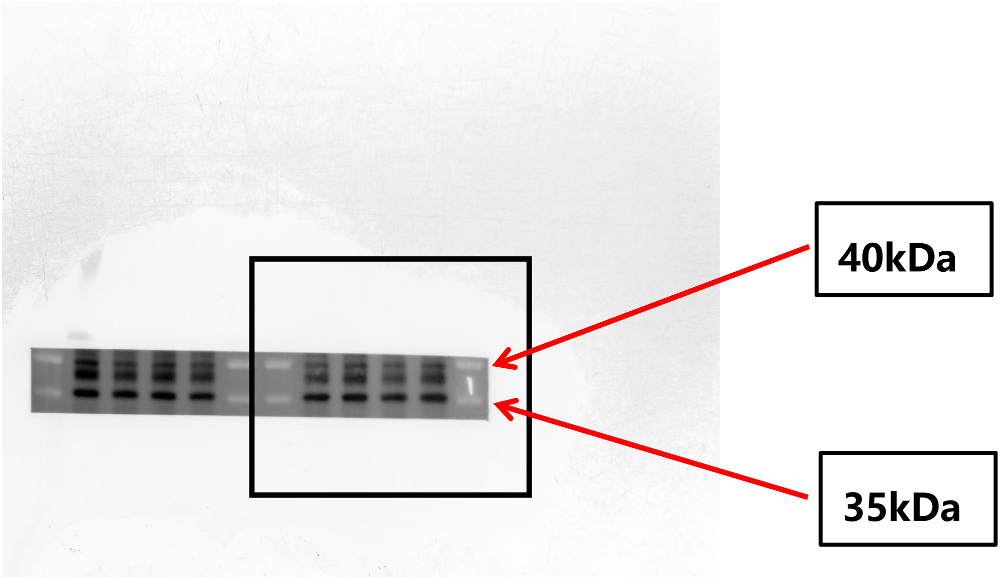

Figure 6

anti-p-PI3K-**85KDa**

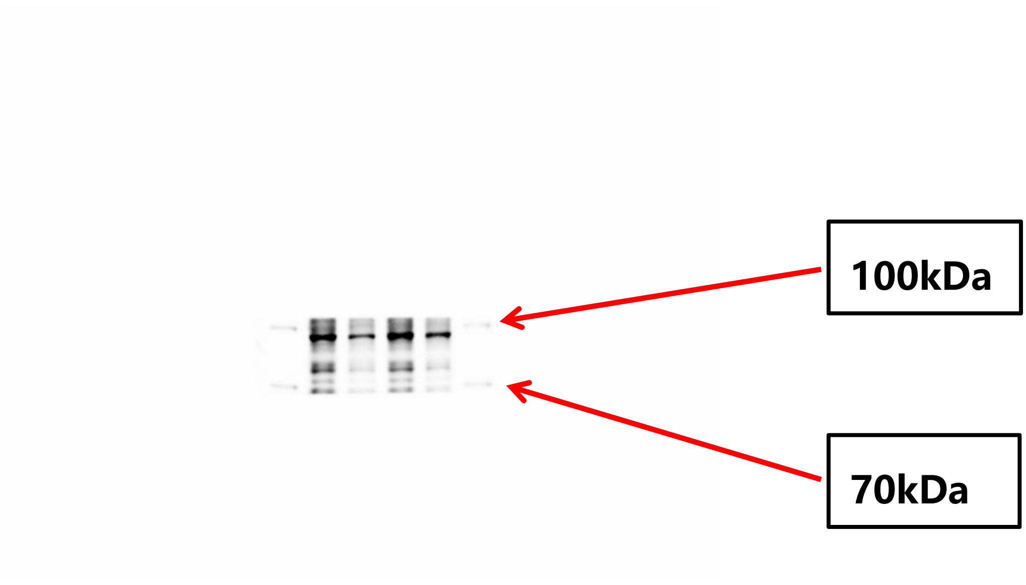

anti-PI3K-**85KDa**

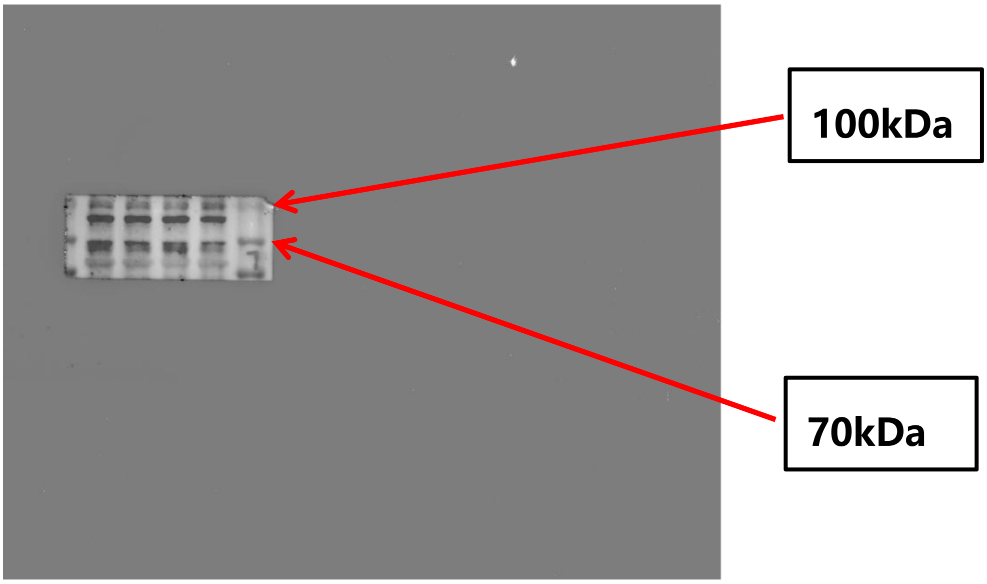

anti-p-AKT-**60KDa**

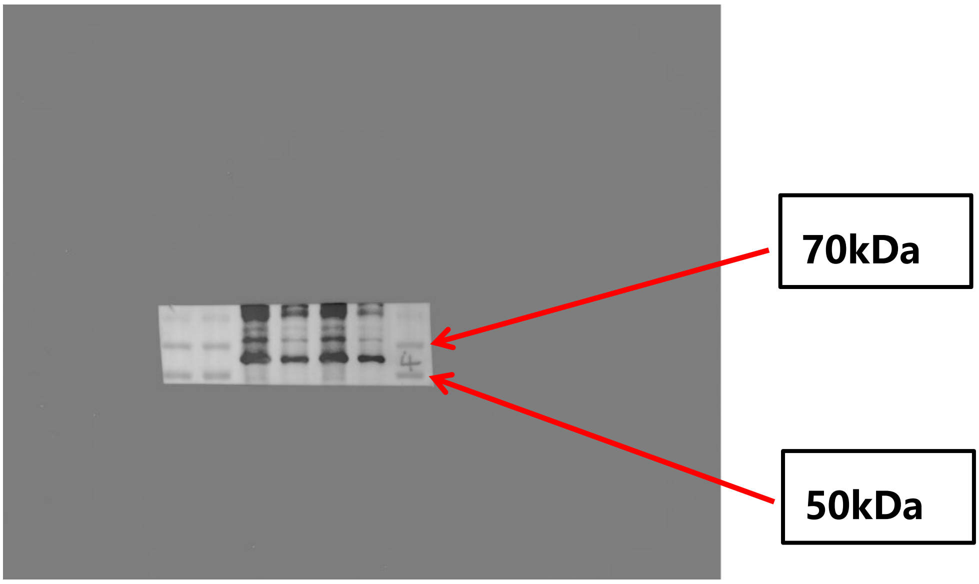

anti-AKT-**60KDa**

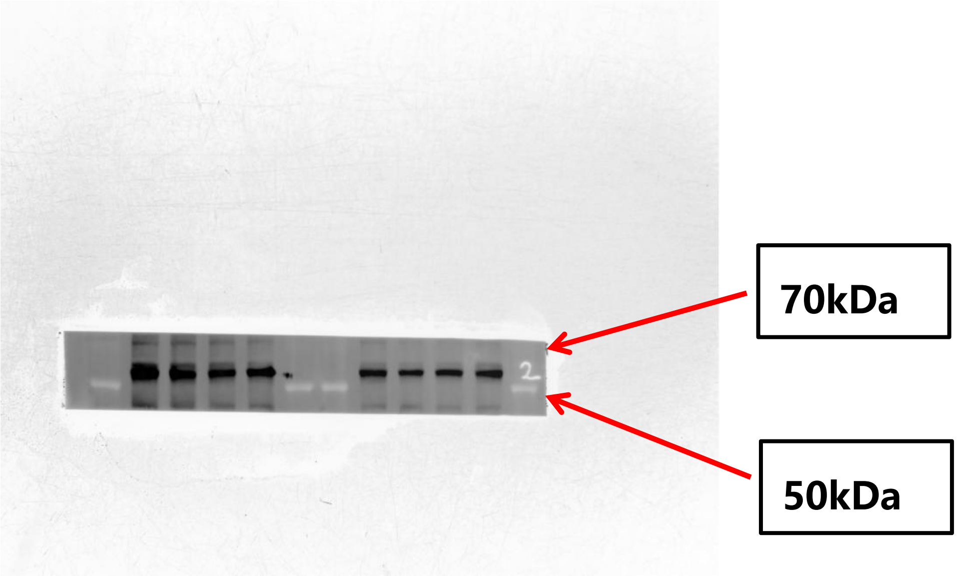

anti-GAPDH-**36KDa**

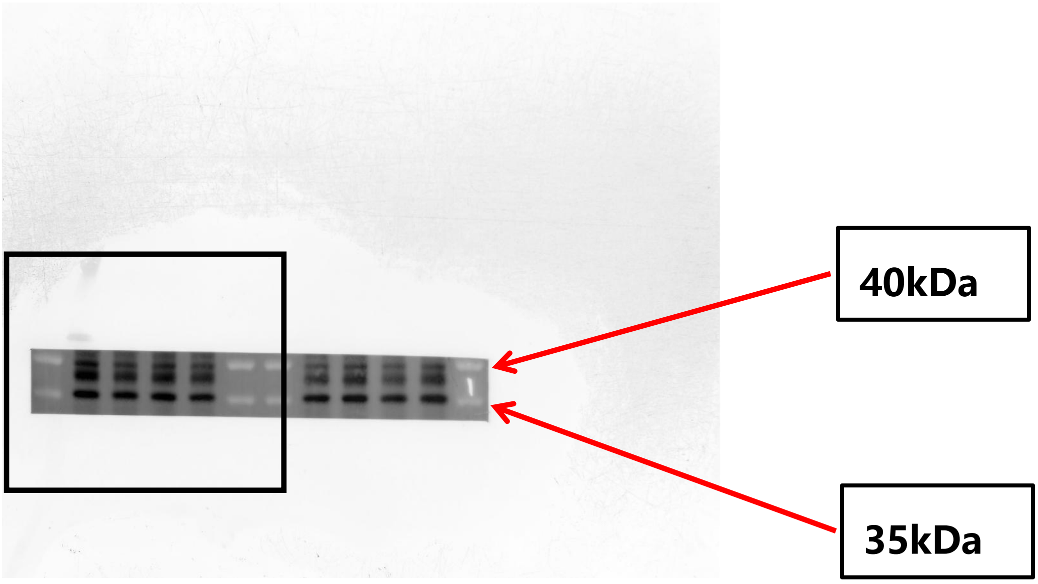

Supplement: Supplementary file 1 — Additional file 1. [file 12957_2023_3128_MOESM1_ESM.pdf]
